# Patient and public involvement in doctoral research: Impact, resources and recommendations

**DOI:** 10.1111/hex.12976

**Published:** 2019-10-15

**Authors:** Nia Coupe, Amy Mathieson

**Affiliations:** ^1^ Manchester Centre for Health Psychology School of Health Sciences The University of Manchester Manchester UK; ^2^ Division of Nursing, Midwifery and Social Work The University of Manchester Manchester UK; ^3^ Present address: Department of Health Services Research University of Liverpool Liverpool UK

**Keywords:** doctoral research, end‐of‐life care, intervention design, obesity, patient and public involvement

## Abstract

**Background and aim:**

Patient and public involvement (PPI) has potential to enhance health‐care research and is increasingly an expectation, particularly for many funding bodies. However, PPI can be tokenistic, which may limit this potential. Furthermore, few studies report PPI processes and impact, particularly in doctoral research studies, which are seldom reported in peer‐reviewed papers. The aim of this paper was to explore the impact of PPI on two health‐related doctoral research studies and identify how PPI could be used meaningfully at this level.

**Method:**

The PPI processes included (a) involvement of two ‘Research Buddies’ who informed the research design and ensured implementation of a booklet intervention was feasible for family carers, (b) data analysis workshops with ‘Research Buddies’ to identify emerging themes from practitioner interviews, (c) public and stakeholder involvement who informed data collection tool design, and the design of an intervention to help people with obesity who attend weight loss groups.

**Findings:**

The application of PPI enhanced both doctoral studies by assisting data analysis; problem solving and improving recruitment rates; improving the usability and appeal of data collection tools and interventions; and developing implementation strategies. Patient and public involvement was considered a rewarding experience for both researchers and PPI contributors.

**Conclusion:**

This paper demonstrates the value of PPI in doctoral research in relation to its impact on research processes, researchers and contributors. We also present recommendations on how PPI could be incorporated into future doctoral research, including resources required, planning PPI processes and involving PPI contributors in all stages of research.

## 1 INTRODUCTION

### 1.1 Background to patient and public involvement in research

Patient and public involvement (PPI) is defined as ‘research being carried out “with” or “by” members of the public rather than “to”, “about” or “for” them’.^1^ For over two decades, researchers have been encouraged to consider patients at all stages of the research process.^2^ A recent concept analysis of PPI in health and social care identified five operational definitions of public involvement, which were undefined involvement; targeted consultation; embedded consultation; co‐production; and user‐led research (Table 1).^3^ These different approaches vary in the amount of public involvement and are a useful starting point to inform and guide appropriate types of involvement at this level, dependent on resources.

**Table 1:** Overview of PPI within both studies in relation to PPI definitions and stage of research

Types of PPI summarized from Hughes and Duffy^3^	Stage 1. Developing and Planning	Stage 2. Data Collection and Analysis	Stage 3. Dissemination
**Undefined involvement** Limited or no involvement			
**Targeted consultation** Public involvement limited to specific requests and tasks, contributors not otherwise involved in the nature or design of the study	**Study A. PPI panel member** Infrequent consultation with an existing panel member with relevant lived experience. Contact made by email/phone for feedback on study documents and research tools, for example protocols, topic guides		
**Embedded consultation** Public contributors with relevant lived experience are consulted with regularly throughout the research cycle, characterized by the regularity and range of methods/people for consultation	**Study A. Consultation with Service Users and Staff** Service user (weight loss) group provided feedback on the first version of the intervention during one‐off meeting. Staff consulted with at various points throughout the research process on the intervention design and content. Contact by phone/email, and one group meeting	**Study B. Research Buddies** Monthly face‐to‐face meetings and occasional telephone contact with two members of the public with relevant lived experience to discuss the progress of the study and provide opportunity to give feedback on research ideas. The ‘Research Buddies’ were also invited to a data workshop, where anonymized transcripts were discussed and initial themes/categories developed	**Study B. Research Buddies** Research Buddies' consulted to ensure implementation strategies and recommendations for future implementation work were acceptable to family carers
**Co‐production** Public contributors with relevant lived experience are involved as members of the research team as researchers/co‐authors, or contribute to key decisions regarding research processes and findings			
**User‐led research** Members of the public with relevant lived experience, academics and practitioners work together systematically across all areas of the research cycle			

Patient and public involvement can strengthen health‐care research by ensuring relevance for patients and is now required in all research funded by the National Institute of Health Research (NIHR) in the UK.^4^ For instance, the NIHR funds 13 Collaborations for Leadership in Applied Health Research and Care (CLAHRCs) across England, which focuses on applied health research and its implementation through partnerships with local organizations. CLAHRC Greater Manchester (GM) research includes themes such as end‐of‐life, wound care and kidney health, and, as an NIHR funded organization, is expected to include and report on PPI (see^5^ for an example of a PPI evaluation within CLAHRC). However, whilst this requirement has been seen positively more generally, it may lead to tokenism, with some research reporting little impact of PPI on the research, and instead paying ‘lip service’ to requirements for funding bodies.^6^

### 1.2 Impact of patient and public involvement

Consensus has emerged regarding some benefits of PPI, such as creation of user‐friendly information and data collection tools, appropriate and effective recruitment strategies, and enhanced implementation and dissemination of findings.^6^ Recommendations are available to help with planning, conducting, reporting and evaluating PPI,^6^, ^7^, ^8^, ^9^ and frameworks have been developed for this purpose such as the GRIPP checklist, superseded by GRIPP2.^10^, ^11^ A recent systematic review identified 65 such frameworks, yet the majority of these were used only by their developers, suggesting limited transferability.^12^ Few studies have evaluated the impact of PPI, potentially due to the lack of robust tools,^13^ and the wide diversity of study/context‐specific PPI aims and approaches.^14^ Whilst it may not be possible to measure the impact of PPI quantitatively, the evidence base could be enhanced by reporting contextual details, aims and available resources.^15^ Furthermore, Greenhalgh et al^12^ question the utility of a ‘one size fits all’ framework for supporting, evaluation and reporting PPI in research, and suggest an adaptable ‘menu of evidence based resources’ (p.1).

### 1.3 Patient and public involvement in doctoral research

Despite the potential benefits of PPI, researchers may be discouraged by the resources required to carry out ‘best practice’ involvement, such as those outlined by Harrison et al^16^. Whilst the recommendations are comprehensive, some activities may be particularly challenging for doctoral researchers. For example, ‘securing funds for compensation’ and ‘appointing an engagement coordinator’ are obstacles for students where funding is limited, and if they are not working as part of a team. In some instances, PhD topics and possibly research questions have been decided prior to the doctoral researcher becoming involved, and they may feel that public involvement is not ‘worth it’, given recommendations to start PPI prior to this point. Having ‘regular face‐to‐face meetings’ and ‘allowing time for relationships to build’ are also challenging within the time constraints of a PhD.

Though there are clear obstacles, PPI is possible at this level, as different levels of involvement are possible at all stages. Indeed, PPI is being carried out to a high level within doctoral research, but with notable exception,^17^ the PPI processes and impact in doctoral research studies are seldom reported in peer‐reviewed papers. Moreover, PPI is sometimes not reported at all because of word count restrictions in journals.^6^

This paper therefore aimed to address a gap in the literature, by presenting different PPI approaches taken in two doctoral research studies and outlining impact on research processes and outcomes. This paper also sheds light on how PPI may be used meaningfully in future doctoral studies in relation to available resources, and instils a good working knowledge of PPI practices at the early career stage. Finally, this paper aimed to answer calls for more ‘user‐friendly tools’ for stakeholder recruitment and evaluation^18^ to encourage researchers – particularly doctoral researchers – to engage with PPI.

## 2 METHODS OF PPI

Two different approaches to PPI were used in the two studies. Whilst the paper's aims are presented above in relation to providing some suggestions and tools for conducting meaningful PPI at doctoral and early career level, we have included the study‐specific PPI aims and approaches to demonstrate impact. The approaches are framed around the conceptual definitions presented by Hughes and Duffy^3^ (Table 1) and reported using the GRIPP2 Checklist^11^ (Appendix D).

### 2.1 Designing a dietary contract intervention for adults with overweight and obesity in low socio‐economic status areas (Study A)

The overall aim of Study A was to design an intervention to improve outcomes for adults with overweight or obesity attending weight loss groups in a low socio‐economic status (SES) area. This population tends to drop out of research earlier,^19^ engage less with^20^ and have poorer adherence rates to weight loss interventions.^21^ The first stage of designing the intervention was a qualitative study, which aimed to identify the particular barriers to lifestyle behaviour change for this population, reported elsewhere.^22^ Following this, the findings were framed within the constructs of the COM‐B model and the intervention was designed following the principles of the Behaviour Change Wheel,^23^ described elsewhere.^24^ The final intervention was a goal‐setting and behavioural contracting booklet, focusing on common problematic dietary behaviours.

#### 2.1.1 Aims of PPI

1. To obtain feedback on the appropriateness of overall PhD research questions and aims.
2. To check readability and suitability of data collection tools throughout the study.
3. To finalize the intervention booklet design, checking appropriateness of content and usability.

#### 2.1.2 Method

The overall method employed in this study is an example of both Targeted Consultation (aim 1) and Embedded Consultation (aim 2)^3^ (Table 1). To meet the first two aims, a CLAHRC team member invited members from their university‐based PPI panel to be involved in the PhD. The already established panel was contacted first as feedback could be sought immediately, thus meeting recommendations to involve contributors as soon as possible. One member expressed an interest by contacting the researcher directly, and both the researcher and members felt they were suitable as they self‐reported as overweight and lived in the same city as the study site. The panel member had also attended training provided by Citizen Scientist. A brief overview of the PhD and what would be required had been circulated during the invitation stage, but no person specification was developed given the panel was already established. The researcher contacted the contributor at various points throughout the doctoral research study, and documents (eg protocols, topic guides) and feedback were shared by email and/or telephone. The researcher asked the contributor specific questions about the documents used in the first qualitative study, as well as more open questions for general feedback on the direction of the PhD as a whole. The researcher also ran through the topic guide with the contributor, ensuring the sensitive wording of any weight and socio‐economic‐related questions. The contributor was paid £75 for half a day's work through submitting expenses forms to the university. This amount had been clearly specified by CLAHRC in their PPI payment guidance. Whilst INVOLVE does not provide a recommended payment rate, they specify that as a minimum, out‐of‐pocket expenses are covered.

To meet the third aim, contributions were sought from members of the public using the service implementing the intervention (service users) and from staff involved in running the service (staff). Involving both groups in intervention development is suggested, given they are ultimately the end‐users,^9^ and are best placed to say if the intervention would be usable and useful in that particular setting. Staff had practical knowledge about what would work within the service/population, and service users have valuable opinions on what they would like or use. Involving a larger group of service users (rather than a ‘Research Buddy’ approach for example) was deemed suitable given the diversity in knowledge and abilities identified in the first qualitative study,^22^ and a more participatory approach was not used given the time limitations. The researcher approached staff already involved in the research, and through the staff identified a suitable service user group to involve. No person specification was advertised. The intervention booklet itself was informed by qualitative research and the wider literature, and groups were arranged once it was developed enough to distribute to the groups. Involving contributors at this stage meant there was something tangible on which people could have an opinion, which was potentially more time efficient. Also at this pre‐feasibility stage, there was still time to make significant changes if required. Two separate meetings took place with these two groups, largely due to the practical difficulties of arranging joint groups:

##### Service user group

The researcher attended an hour‐long weight loss group, run by the local authority within a low SES area. On that particular week, the usual staff member was absent, and the service invited the researcher to use the session to gather informal feedback on the first version of the intervention booklet. The booklet was introduced and distributed to ten service users, followed by some time allowing them to use the booklet. The remainder of the session was spent looking through responses and collecting feedback in small groups. The researcher used open prompts relating to usability and suggestions for improvements. Those who contributed were given a £20 Love2shop voucher for their time, which was provided by the researcher's funder (NIHR CLAHRC GM). As no contact details were taken, it was not possible to provide feedback to this group regarding changes made as a result of the meeting. However, a lay summary of the PhD as a whole is planned to be made available to the service, which will describe the PPI and acknowledge its impact on the research.

##### Staff group

To gain feedback from the deliverers' perspective, a one‐off meeting with staff members who would be delivering the booklet was arranged. Three weeks prior to the meeting, the booklet was circulated by email to eight staff members who had been involved in the initial research study. Three members of staff attended, and the remaining five, who could not attend, were invited to provide feedback by phone or email. In the meeting, staff members were asked to provide their initial impressions of the booklet. Each member was then asked to provide any further suggestions to improve the booklet's content, usability and appeal. The meeting, conducted in a central office within the local authority, lasted one hour. Staff contributors were sent the finalized booklet by email to see the changes made as a result of the meeting. Payments in exchange for staff's time were not permitted given staff were employed by the local authority. Staff were invited to provide feedback on their experience of PPI by email following final data collection (Appendix A).

The booklet was also emailed to the PPI panel member for any final points, to include input from someone outside of the service in case there was too much assumed knowledge. The PPI panel member was sent a £20 Love2shop voucher. As with the service user group, no feedback was given regarding changes made, but a lay summary will be sent.

The researcher took written notes throughout both sessions and from the email to ensure any suggestions could be incorporated into the next iteration of the booklet. Notes were recorded using Appendix C.

### 2.2 Supporting family carers in providing care at home (Study B)

The overall aim of this study was to explore and evaluate the processes involved in the implementation and adoption of an evidence‐based intervention for family carers (the ‘Caring for Someone with Cancer’ booklet)^25^ in community nursing. Little is known about the work required to implement evidence‐based practice within community nursing,^26^ which is challenged by a lack of consistency in the terminology used.^27^ This study therefore contributed to the evidence base by (a) exploring how the booklet intervention had been used within a large NHS Community Trust and (b) utilizing Participatory Action Research principles and Normalization Process Theory^28^ to implement the booklet intervention in four sites in one city in England, UK. A qualitative case‐study design was adopted, combining observations with semi‐structured interviews and focus groups with practitioners.

#### 2.2.1 Aim of PPI

1. To obtain the perspectives of family carers on the implementation work and findings throughout.
2. To ensure implementation strategies and recommendations for future implementation work are acceptable to family carers, as ‘end‐users’ of the booklet intervention.

#### 2.2.2 Method

The overall method employed in this study was Embedded Consultation given there was regular consultation throughout the research process.^3^ The role of public contributors within this study was developed by the researcher after discussion with a member of an existing PPI panel. The researcher decided that two ‘Research Buddies’ would be recruited to give their perspective. The benefit of this approach was that the opinions and advice of the ‘Research Buddies’ could be sought flexibly throughout the study; ensuring implementation of the booklet was feasible for family carers, in addition to the nurses, who were the primary participants. The ‘Research Buddy’ approach was also the most feasible in terms of resources available.

To recruit the ‘Research Buddies’, an invitation and role description (Appendix B) were devised by the researcher and readability checked by staff at a carer centre in the study area. The invitation and role description were advertised at the carer centre, online and through a PPI group at the University of Manchester. Three carers volunteered: one having seen the advertisement online and the other two at the carer centre. The researcher met individually with the volunteers to explain the role, and it was mutually agreed that two carers would be consulted individually on a monthly basis, during the data collection, analysis and dissemination stage of the research process, with the third volunteer opting to be a study participant rather than a ‘Research Buddy’. Both ‘Research Buddies’ were female and had experience of caring for their husbands, who had cancer, at home towards the end of life. The ‘Research Buddies’ also volunteered at a local hospice.

The researcher had regular contact throughout the study with the two ‘Research Buddies’, both face to face and by telephone, to discuss the study's progress and garner their perspective as former carers. The ‘Research Buddies’ were also invited to take part in data workshops held at the university, lasting approximately two hours. Prior to data workshops, the data analysis approach was explained, and the ‘Research Buddies’ were asked to provide their feedback on anonymized transcripts of interviews with nurses and nursing home staff, to explore what it meant to them as a family carer.^29^ The ‘Research Buddies’ were reimbursed for their time and given a choice of payment of cash or Love2shop vouchers. ‘Research Buddies’ were given £20 per hour of their time, provided by the researchers’ funder (NIHR CLAHRC GM).

At the end of the study, ‘Research Buddies’ provided feedback on their experience of PPI face to face using the evaluation questions provided in Appendix A. The ‘Research Buddies’ were given questions prior to the evaluation meeting and asked to reflect upon their experience in preparation. With the ‘Research Buddies’ consent, the evaluation meetings were audio‐recorded, for use by the researcher. The ‘Research Buddies’ were encouraged to provide candid feedback and asked to answer questions regarding both negative and positive aspects of their involvement (Appendix A). Finally, the researcher provided information regarding other involvement opportunities and established PPI groups within the university.

## 3 FINDINGS

Our findings are framed around the two main impacts the PPI had on the studies: impact on research process and impact on researcher and contributor. We have included some tools in the Appendices, which researchers can adapt to use to assist recruitment and evaluation of public contribution in their studies. The authors found these tools helpful in their doctoral studies and are thus supported by an evidence base.

### 3.1 Impact on research processes and outcomes

An overview of the changes made as a direct consequence of PPI feedback within both studies is presented in Table 2, alongside which aims these addressed.

**Table 2:** Impact on research processes and outcomes within both studies

Study/Contributor(s)	Impact on research process and outcomes
Study A	
PPI panel member	Patient information sheets/consent forms – improved readability, ensuring sheets were appropriately informative by identifying and removing unnecessary information and jargon, where possible (aim 2) Wording of sensitive topic guide questions and study title – ‘low socioeconomic status area’ was simply change to the ‘[target city] name’ (aim 2) The PPI panel member also read the PhD overview and qualitative study protocol, but had no comments to make (aim 1)
Service user group	Layout and content of the booklet in terms of usability & appeal – making it more obvious that only one goal should be selected, choice of food images, making goals colour coordinated for ease of navigation. This was particularly important given the issues around engagement in this population, and the language and literacy barriers identified in the initial qualitative study^24^ (aim 3)
Staff	Content of the booklet – adding a lifestyle goal relating to increasing water consumption, ensuring included guidelines around fat and salt intake were correct and in line with the service's recommendations (aim 3) Design and content of the booklet, including usability and appeal – changing images of food that might be more familiar to the population, adding easy to follow recipes (aim 3)
Study B	
Research buddy	Offering potential solutions when faced with recruitment problems, such as gaining access to, and recruiting additional sites, when a site withdrew from the study (aim 1) Developing emerging themes, by drawing on their experience, for example the categorization of family carers by community nurses and the interactional work which may have an impact on whether or not the booklet is delivered (aim 1) Offering alternative explanations to themes identified in the data, by reading anonymized transcripts and participating in data workshops (aims 1 and 2)

### 3.2 Impact on researcher and PPI contributors

#### 3.2.1 Study A

Overall, the researcher felt the PPI activities had contributed to increased skills and confidence during the doctorate. For example, from organizing PPI activities, leading meetings and making changes following feedback, the researcher felt they increased their skills in organization, communication, partnership building and use of lay language. The latter was particularly useful in developing an intervention that needed to be mindful of language and literacy.

No feedback was obtained from service users following the group session, which was an oversight. Feedback would have contributed to the overall evaluation of the PPI, but evaluation had not been planned at this stage, and no contact details were taken from these contributors for follow‐up. Inviting staff contributors for feedback was easier given the on‐going contact with them throughout the doctorate. As such, two of the three staff contributors who provided evaluation feedback by email. Both stated they would be happy to be involved again and enjoyed the experience. One staff member highlighted that they appreciated seeing the changes made from their involvement:

I was given opportunity to feedback on the booklet, what I thought would work and what wouldn't and [name of researcher] welcomed any suggestions I made. I enjoyed being able to use the booklet and see how it worked for clients and then being able to see my suggestions used in future booklets

Staff stated that they had not necessarily learned anything, but had a new appreciation of the work involved in research. Importantly, they did not feel that there had been any barriers to being involved, particularly as the researcher worked around the staff members:

[name of researcher] was very accommodating, always worked meetings around my work diary and more often than not, she travelled to me, so I don't feel there were any barriers to overcome

#### 3.2.2 Study B

The ‘Research Buddies’ reported only positive aspects to their involvement. In particular, a sense of contributing to society by sharing their experience was identified as beneficial, as was the partnership's reciprocal nature. That is, the researcher and ‘Research Buddies’ learnt from each other. Specifically, the ‘embedded consultation’ approach allowed the researcher to explore issues important to family carers and gain insights into the booklet's implementation from a family carer perspective, which could not have been gleaned from supervisors or academic resources. Furthermore, by collaborating with the ‘Research Buddies’ throughout the study, the researcher developed skills in written and oral communication for lay audiences.

Sharing the pitfalls and successes of the research was a positive experience for both the researcher and the ‘Research Buddies’. For one Research Buddy, the ‘sharing and caring’, as a result of her involvement, was enjoyable, as she discusses below:

I've enjoyed sharing with you [the researcher], sharing information, thoughts, plans, all the sharing things I've enjoyed. I've enjoyed caring about you and what's happened to you, [laugh] and your disappointments, and the stress…

This was also enjoyable for the researcher, who found the meetings a source of support, in addition to meeting with supervisors. The ‘Research Buddies’ would listen, sympathize, relate problems the researcher had encountered to her experience of caring for someone, and offered potential solutions. Furthermore, the ‘Research Buddies’ offering support and relating problems to their own caregiving experience improved the researcher's understanding of the availability and workload of community nurses, particularly family carers’ interactions with District Nurses and potential implementers of the booklet, that is health‐care professionals who have opportunities and time to provide more support to family carers.

### 3.3 Researcher reflections

Though the experiences were generally positive, we identified some potentially negative or challenging aspects of PPI within our studies. For Study A, using two approaches (panel member/face‐to‐face groups) worked well as staff were too busy to be involved in both aspects, and the feedback required fitted well with the experience and knowledge of the respective contributors. However, whilst there were benefits to involving staff as researchers and as contributors, such as enhanced rapport and overall engagement with the research, there was a lack of clarity on these two distinct roles. This became apparent in the feedback, which for one staff member focused using the booklet in their groups, rather than their contribution towards its design. The roles were explained at various points throughout the PhD and highlighted when asking for feedback on their PPI experience. The researcher relied on established relationships with staff from the earlier stages of research in recruiting staff as contributors. However, as previously mentioned, the researcher did not advertise or provide a person specification for the PPI role, which in hindsight could have clarified the two roles for staff. Furthermore, had the PPI meeting been arranged and clearly labelled as one larger PPI event for service users and staff, the roles and purpose may have been clearer. However, there were benefits to having separate groups, including ease of arranging, reduced resources and avoiding potential difficult situation of paying only half of the attendees, possibly making the staff feel as though their time and input was less valuable.

Although not necessarily planned as such, all contact with the PPI panel member in Study A was by phone and email. This had clear benefits in terms of being time efficient, not having to wait for availability to meet. However, the possible drawback was that not meeting face to face may have hindered the relationships and rapport building. Though a lay summary is planned for the end of the PhD, providing feedback in a more timely manner may have resulted in further clarification or other changes, and help the contributor develop skills as a PPI panel member. Furthermore, using phone/email had clear benefits of being low resource which may be particularly useful within time and budget limitations of doctoral research (and still resulted in some impact), including one or two face‐to‐face contacts may have enhanced this.

The researcher considered PPI particularly useful given they were not from the same socio‐economic background as the target population, specifically in reducing potential preconceptions about the factors involved and which elements of the booklet may be challenging.

For Study B, it was often necessary to limit the involvement of the ‘Research Buddies’ due to resources and time constraints. For example, one ‘Research Buddy’ was keen to involve other sites and extend recruitment to include patients, which was not feasible for this doctoral study. Whilst the ‘Research Buddies’ enthusiasm and willingness to dedicate time to the study were largely positive and a testament to the relationship built, it was important to match expectations, to ensure the experience was mutually beneficial and that the project was completed on time.

Though PPI was supported by both supervisory teams, it was not an expectation from an organizational perspective to include PPI. Furthermore, though PPI within NIHR funded research is required, it was not necessarily an expectation within the PhD research, particularly for Study A which was not attached to a CLAHRC ‘theme’. Both students were selected through CLAHRC to attend NIHR training focusing on PPI within the first year of their PhDs and were made aware through their funding body that both funding, and a PPI panel based at the University were available. This was a clear facilitator to PPI, although the exact budget available was unknown to the researchers. Furthermore, both students had attended university training on setting up PPI groups and had some experience of PPI having worked as researchers within CLAHRC GM and utilizing an established PPI panel. However, this experience was largely retrospective evaluation and/or engagement; therefore, the PhD provided an opportunity for planning PPI prospectively. Overall, though it was well supported, PPI in both studies was student‐led and would have unlikely occurred without the funding and previous experience. This highlights the importance of the need for supervisors, and funding bodies to ensure students are aware of PPI, ideally providing training and raising awareness of the resources available during their planning stages.

## 4 DISCUSSION

**Figure 1:**
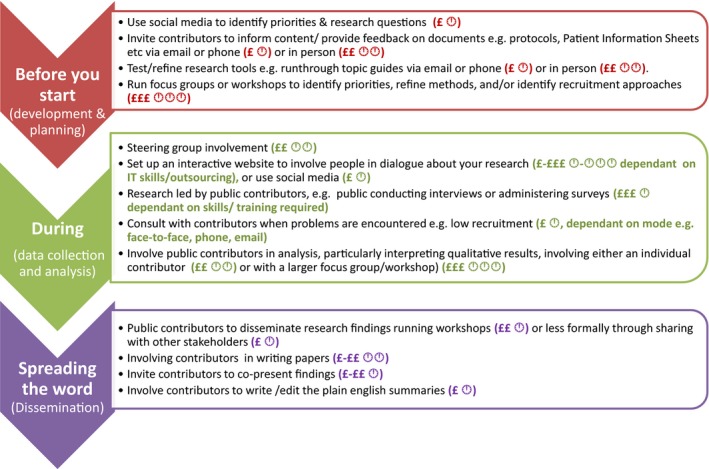
Suggestions for PPI activity during doctoral research in relation to available resources. Key: £ lower cost ‐ £££ higher cost, 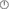, less time required ‐ 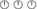, more time required

This paper outlines why PPI within postgraduate research is important, drawing on examples from doctoral studies in both end‐of‐life care and public health intervention development. For Study A, PPI resulted most notably in important changes to the intervention booklet's content, such as including a water consumption goal, and in ensuring the appropriateness of the qualitative topic guide. For Study B, PPI ensured the feasibility of the intervention from an otherwise overlooked family carers’ perspective and enhanced the development of qualitative themes. Results highlight that PPI at this level not only impacted the quality of research (eg improving usability and appropriateness) and research processes (eg recruitment), but also positively impacted the researchers and contributors themselves. Contributors valued being involved, contributing to society and seeing their suggestions develop the research. The researchers felt the contributors provided valuable insight into their research, and their experience of PPI resulted in developing important early career skills such as communication and building partnerships. Building on the success and lessons learnt from the presented studies and informed by the literature^9^, ^30^ we have provided an overview of possible approaches to conduct meaningful PPI at postgraduate level in relation to resources and stage of research (Figure 1), along with recommendations. Together, this adds to the guidance around how to conduct PPI which is currently lacking,^18^ particularly for those conducting PPI at doctoral level, or for those who may be new to it.

Doctoral programmes should ensure doctoral researchers are sufficiently experienced and qualified to become the next wave of leading researchers. Our findings support the inclusion of PPI in doctoral training, if it is to become an essential (and well‐conducted) part of research. Students should therefore be supported financially and encouraged by their institutions and supervisory teams. In this instance, the doctoral researchers had funding available through the NIHR to support PPI costs and thus were supported to include PPI in their research. Ensuring sufficient resources therefore is a particularly important consideration at the institutional level, to allow the calculation of PPI funding into the doctoral research budget. However, not all doctoral students will have the resources for co‐production or user‐led type PPI. This paper highlights that doctoral researchers can still conduct meaningful PPI, even with lower level involvement or minimal resources (Table 2).

As discussed, the ‘Research Buddies’ in Study B reported their involvement to be valuable, contributing to both the progress and quality of the research. This was also reported by Mann and colleagues^31^ when analysing the perception of both PPI contributors and researchers regarding the process and impact of PPI within a randomized controlled trial. Patient and public involvement contributors felt valued and claimed interactions with researchers were enjoyable.^17^, ^31^ The positive impact on those involved is not limited to cancer and end‐of‐life care research; indeed, benefits have been reported across a broad range of health‐ and social care studies, including empowering individuals and improving researcher understanding.^6^ Taken together, this suggests that meaningful PPI is possible, even in sensitive research areas such as cancer and palliative/end‐of‐life care.

Whilst there are many benefits of including PPI in the research process, there are some potential issues for both the contributors and the researchers, which must be acknowledged. In end‐of‐life care and cancer research, it is important for those conducting PPI to be suitably equipped in dealing with distress, having appropriate resources and processes in place. However, this was not an issue encountered in Study B, as discussed in the findings sections. Similarly, in another study exploring the experience of prostate cancer patients, all members of the PPI group claimed being asked to participate gave meaning and value to an otherwise unpleasant experience.^17^ Involving family carers as public contributors in end‐of‐life care research may also be an issue encountered by researchers, and it is important to note that both ‘Research buddies’ in Study B were retired and former carers meaning they had fewer commitments.

Running public involvement events can be time and resource intensive for both the researcher (possibly delaying research further) and the contributors. Neither studies were delayed by the included PPI work, in fact PPI within Study B helped enrol another site when one site withdrew, thus reducing time spent on recruitment. However, as discussed, the PPI work may have delayed Study B if the researcher had not reviewed the level of involvement and prevented one Research Buddy recruiting additional sites and patients.

One issue within Study A was that staff was involved as both participants and contributors, which resulted in some being unclear with regard to the two different roles, rendering obtaining feedback on the PPI work difficult. Though this approach of combining qualitative research and PPI with hard to reach populations has been used successfully elsewhere,^32^ future researchers should ensure that those involved are clear on the distinction between roles from the outset. An alternative approach in this study may have been not to include staff members within the research aspect, but to include them as stakeholders within more of a co‐production approach (see Table 1). However, given that staff had limited time, particularly following cuts to their service, this may not have been feasible.

A possible expectation of establishing relationships with public contributors is their continued involvement in research. At doctoral level, this may not always be possible, given that postdoctoral researchers often move on to different geographical and/or research areas; doctoral researchers should ensure that in addition to having clear goals at the beginning of the research,^3^ there should be clear expectations regarding what will happen at the end of the research process. This was a particular issue within Study B, as highlighted in the findings. If stakeholders express a wish to continue to contribute, doctoral researchers can signpost to other opportunities within institutions, or other places where such roles are advertised.

Hughes and Duffy^3^ argue that reporting PPI work separately from main outcome papers may contribute to its tokenism. In this particular instance, outlining the impact of PPI separately from the main outcome papers was important, to highlight the practicalities and potential impact of PPI at this level, particularly given word count restrictions, as previously noted. Reporting PPI impact separately may also make identifying such evidence easier and making for more detail regarding the PPI activity available to others, given the sparsity of evidence identified.^18^

## 5 IMPLICATIONS AND RECOMMENDATIONS FOR CONDUCTING PPI AT POSTGRADUATE LEVEL

We have demonstrated that conducting impactful PPI with limited resources is possible at this level, highlighting that PPI should not be dismissed on grounds of cost or time. University policies should ensure PGR students are provided with training and resources, as well as supervisory (or other) support to include PPI, both for the quality of the research and the development of researcher skills. An evidence gap exists in relation to how best to facilitate involvement with those with time commitments (eg staff, current carers) and hard to reach groups, for example through utilizing technology such as Skype.

Given the effect PPI had on the research presented in this paper, we have combined our experience of PPI at this level with the literature and recommendations currently available.^30^ Recommendations summarized in Table 3 build on suggestions presented in our blog,^33^ and in Figure 1 are presented in relation to both the stage of doctoral research and resources available to the researcher.

**Table 3:** Recommendations, references and tools for conducting PPI within doctoral research

Recommendations	References and links	Tools provided
(1) Consider your aims		
What is the purpose of the PPI and what you would like to achieve from it, for example do you need to clarify and further refine your research question, or would the data collection benefit from some expert input? Having clear aims will help avoid tokenism, and will show clear purpose for your work	You can access INVOLVE's library of past and current work for ideas and inspiration,^34^ and the definitions outlined by Hughes and Duffy^3^ may be useful to consider the type of PPI	
(2) Consider your resources		
Is funding available to you to support PPI costs? How much time you have (within your current study/PhD)? Even if your resources are low, activities can still be high impact and/or not tokenistic	Recommendations in Figure 1 should be used in accordance with public involvements standards, such as those laid out by INVOLVE^35^	Figure 1
(3) Seek advice		
Speak to someone with experience and knowledge of PPI. Many universities will have someone working solely on PPI/public engagement. If not, find out if any colleagues have any expertise and arrange to meet with them Identify if there is a PPI group already running that you could work with‐ this can save some time for you	If no PPI panel exists, try relevant charities, and/or advertise online (eg (http://www.peopleinresearch.org/) or using social media, local libraries and community centres	
(4) Record & report		
Keep track of all PPI activities to report impact. Think about how you will report your PPI work. All views must be valued, and if in some instances feedback cannot be acted upon (eg if not feasible) then this should be fed back, so contributors feel heard. Keep notes of positive and negative effects of PPI on your research;	It may be helpful to identify a framework to assist you in how you will evaluate and report your own PPI work, and what to record, for example see Greenhalgh et al's systematic review of PPI frameworks^12^	Appendix A, Appendix B, Appendix C
Some ways to record impact include: Impact on researcher – keep a reflective diary of the activities Impact on contributor – invite feedback throughout the process if possible, and at the end Impact on processes – keep a record of each activity, clearly recording what exactly was done, when, what feedback/input was received, and what changed as a result	Consider using the GRIPP2^11^ checklist), which will help ensure your work is contributing towards the growing PPI literature	Appendix D

## 6 CONCLUSION

This paper has outlined why PPI at postgraduate level is important. Patient and public involvement does not have to result in huge changes to be meaningful; through small changes, our PPI resulted in improved quality, and importantly was meaningful to our contributors. Learning to conduct meaningful PPI at postgraduate level is also important in shaping future generations of researchers, thus impacting on the quality of future research. For doctoral researchers looking to conduct meaningful PPI, we have summarized our recommendations and suggestions on how this can be achieved at different stages, dependent on available resources. To ensure PPI at the doctoral level is non‐tokenistic, it must be supported and funded sufficiently, and therefore costed into doctoral programmes, and encouraged at both the supervisory and institutional level.

## CONFLICT OF INTEREST

The authors declare no conflict of interest.

## Data Availability

The data that support the findings of this study are available from the corresponding author upon reasonable request.

## APPENDIX A

### Stakeholder Evaluation Questions

1. Describe your experience of being a [insert stakeholder engagement term].
2. What have you enjoyed or liked about your experience of being a [insert stakeholder engagement term]?
3. What are the benefits to having stakeholders involved in this project and similar studies?
4. How has your involvement improved the research?
5. What have you learnt?
6. What have you not enjoyed or have not liked about being a [insert stakeholder engagement term]?
7. What could have been done differently or could have been improved?
8. What are your thoughts on the practicalities of being a [insert stakeholder engagement term]? (eg travelling; attending meetings; doing work at home)
9. Did you feel sufficiently supported/trained to be involved in the study? Why/why not/how could it be improved?
10. Would you be willing to be involved in research in the future?
11. What can researchers do to encourage other stakeholders to be involved in research?

## APPENDIX B

### Role Description for [insert stakeholder engagement term]

**Study Title: **XXXXXXXXX

**Background:**

[Brief introduction to study and researcher]

***What is the purpose of the study?***

[Describe why the research is important, aims and objectives etc.]

***What is a [insert stakeholder engagement term]?***

[Briefly describe role and responsibilities. Clarify that volunteers will not be a participant or subject of the research, and state that their contribution would be highly valued]

**Qualifications:**

You do not need ANY formal qualifications to be a XXXXX. We are looking for people that:

[Bullet points e.g. *‘Do not work professionally in health and/or social care roles’, live in [Area], can communicate freely in English, as we cannot offer interpreters at present*].

**Experience and Personal Qualities:**

[Briefly describe the experience and personal qualities you are looking for the stakeholder e.g. *experience of caring for people with XXXXX, ‘willing to listen to other people's views and respect others' opinions, even if they disagree with them’*].

**Your Responsibilities:**

As a [insert stakeholder engagement term]:

[Bullet point specific responsibilities].

**Our Responsibilities:**

[Bullet point the researcher's responsibilities e.g. *‘XXXXX will be your named contact, XXXXX will meet you to discuss the role and will answer any questions you may have’, ‘most meetings will be held at XXXXX', 'support and guidance on XXXXX will be provided'*].

**Duration of Role:**

The project will run from XXXXX to XXXXX. We would like the [insert stakeholder engagement term] to be involved until XXXXX. We understand however, that unpredictable events may occur which means you have to withdraw from the project.

**Payment and Expenses:**

[Provide details on travel expenses and payment]

Who is organising the study?

XXXXX is the lead researcher and is responsible for the day‐to‐day running of the study.

XXXXX is being supervised by:

[Insert supervisor contact details].

[Insert funding statement].

**Contact details:**

For more information please contact: XXXXX

## APPENDIX C

### PPI activity record

**Table:**

Impact of PPI on research processes & outcomes				
Date	What we did	What was said	What we changed	What was the impact (positive & negative)
	*eg Sent a copy of the topic guide to the member of the panel by email, asking for general feedback and specific feedback on wording of question 8. Phoned them one week after at an agreed time*	*Panel member suggested XYZ*	*Question 8 was brought forward and wording changed from X to Y*	*Topic guide flowed better, and I was not uncomfortable asking the question during the interview*

## APPENDIX D

### GRIPP2 Short Form

**Table:**

Section and topic	Item	Reported on page No
1. Aim	Report the aim of PPI in the study	4&6
2. Methods	Provide a clear description of the methods used for PPI in the study	4‐7
3. Study results	Outcomes—Report the results of PPI in the study, including both positive and negative outcomes	7‐10
4. Discussion and conclusions	Outcomes—Comment on the extent to which PPI influenced the study overall. Describe positive and negative effects	11‐14, Table 2
5. Reflections/critical perspective	Comment critically on the study, reflecting on the things that went well and those that did not, so others can learn from this experience	10‐11
